# The Effect of Differential Repeated Sprint Training on Physical Performance in Female Basketball Players: A Pilot Study

**DOI:** 10.3390/ijerph182312616

**Published:** 2021-11-30

**Authors:** Jorge Arede, Sogand Poureghbali, Tomás Freitas, John Fernandes, Wolfgang I. Schöllhorn, Nuno Leite

**Affiliations:** 1 Department of Sports Sciences, Exercise and Health, University of Trás-os-Montes and Alto Douro, 5001-801 Vila Real, Portugal; nleite@utad.pt; 2 School of Education, Polytechnic Institute of Viseu, 3504-501 Viseu, Portugal; 3 Department of Sports, Higher Institute of Educational Sciences of the Douro, 4560-708 Penafiel, Portugal; 4 Institute of Sport Science, Otto-von-Guericke-Universität Magdeburg, 39104 Magdeburg, Germany; sogandpoureghbali@gmail.com; 5 UCAM Research Center for High Performance Sport, Catholic University of Murcia (UCAM), 30107 Murcia, Spain; tfreitas@ucam.edu; 6 NAR-Nucleus of High Performance in Sport, São Paulo 04753-060, Brazil; 7 Faculty of Sport Sciences, Catholic University of Murcia (UCAM), 30107 Murcia, Spain; 8 School of Sport and Health Sciences, Cardiff Metropolitan University, Cardiff CF23 6XD, UK; jfmtfernandes@hotmail.co.uk; 9 Institute of Sport Science, Training and Movement Science, University of Mainz, 55122 Mainz, Germany; schoellw@uni-mainz.de; 10 Research Center in Sports Sciences, Health Sciences and Human Development, CIDESD, University of Trás-os-Montes and Alto Douro, 5001-801 Vila Real, Portugal

**Keywords:** jumping, sprinting, change-of-direction, fluctuations, bilateral asymmetry

## Abstract

This pilot study aimed to determine the effects of differential learning in sprint running with and without changes of direction (COD) on physical performance parameters in female basketball players and to determine the feasibility of the training protocol. Nine female basketball players completed 4 weeks of repeated sprint training (RST) with (COD, *n* = 4) or without (NCOD, *n* = 5) changes of direction. A battery of sprints (0–10 and 0–25 m), vertical jumps (counter movement jump (CMJ), drop jump, and single-leg CMJs), and COD tests were conducted before and after intervention. NCOD completed two sets of ten sprints of 20 m, whereas COD performed 20 m sprints with a 180 degree turn at 10 m, returning to the starting line. Before each sprint, participants were instructed to provide different fluctuations (i.e., differential learning) in terms of varying the sprint. Both groups had 30 s of passive recovery between two sprints and 3 min between sets. A significant effect of time for the 0–10 m sprint, CMJ, and single leg-CMJ asymmetries were observed. Adding “erroneous” fluctuation during RST seems to be a suitable and feasible strategy for coaches to enhance physical performance in young female basketball players. However, further studies including larger samples and controlled designs are recommended to strengthen present findings.

## 1. Introduction

The success of team sports depends, to a large extent, on the physical abilities of a player but also on the technical and tactical skills [1]. In team sports (e.g., basketball), repeated bouts of high-intensity activities (i.e., sprinting, jumping) are interspersed with periods of low-to-moderate activity or passive recovery [1]. Moreover, the physical demands are complex and challenge athletes to have highly and simultaneously developed speed, agility, strength, power, and endurance qualities [1]. Therefore, it seems plausible that practitioners working within team sports design training regimes that adequately match the specific team sport requirements.

Players’ ability to perform repeated sprints is one of the critical determinants of team sports performance [2], including in basketball [3]. In team sports, sprinting activities correspond to a small percentage of total distance covered or number of activities [4,5]; however, short sprint activities are intercepted by a short period of time (i.e., every 21 to 39 s), for example, in basketball [6]. In this regard, although the total distance covered during competition has not changed over the years, the requirement for high-intensity running and longer sprint distances has increased [7]. Consequently, it is astute that practitioners develop methods of enhancing sprint and repeat sprint ability (RSA) in team sports athletes. Moreover, since sprints in these contexts are not exclusively straight-line, it is considered beneficial to prepare athletes to sprint in different directions, challenging the technical model.

Various methods are used to develop sprint and change of direction (COD) performance, including assisted and resisted sprinting techniques, resistance training, plyometrics, and rotational flywheel training [8,9,10]. A comparison between RSA- and COD-training revealed comparable performance improvements in soccer-relevant physical performance factors [11]. However, evidence suggests that sprints with COD and short shuttle runs are more demanding than linear sprints [12], resulting in higher cardiorespiratory involvement and blood lactate accumulation [12]. Moreover, sprints with COD include deceleration and acceleration efforts, creating heightened metabolic and mechanical demands needed to overcome inertia and rapidly generate propulsive forces in a new direction [13]. This might evoke more significant stimuli in performance components associated with neuromuscular factors such as jumps, sprints, and repeated sprint performance [14]. However, sprints and sprints with COD are performed during the game in unpredictable situations and in different contexts, so it is warranted to employ training methods that promote both the ability to adapt and the development of the necessary physical capabilities.

From a dynamical systems perspective, fluctuations play a key role in the adaptation of living systems when transiting from one stable state to another [15]. On the basis of bio-mechanically identified, different levels of fluctuations in several highly automatized movements [16,17], the differential learning (DL) model proposed a concrete practical transfer of the system dynamics approach to gross motor movements by utilising and actively increasing fluctuations in an athlete’s movement learning process [18]. In contrast to the traditional training strategy, where fluctuations are considered errors that must be minimized, the DL approach considers the fluctuations in moving systems as potential sources and necessary for learning. In an analogy to artificial neural nets that are modelling phenomena of real neurons [19], an increase of noise during the learning phase fosters the process of interpolation and, as a consequence, allows the system a better performance in the subsequent application phase [15]. Evidence for better performance by interpolation compared to extrapolation in natural neuro-motor systems was provided by a study by Catalano and Kleiner [20]. The experiment shows significantly better performance when testing within the trained range than when testing outside. Accordingly, the DL theory recommends increasing the range already during training to increase the probability of being able to interpolate in case of emergency or already during the next movement alone and thus to be able to react more adequately to the new elements that will surely come. Fluctuations can be increased along a neuromechanical strategy [18] in which the angles, angular velocities, and angular accelerations of all joints or their rhythms are varied, since each biomechanical variable can be associated with a physiological proprioceptor that provides the information necessary for training to the central nervous system for reorganization. Fluctuations also can be increased by all kinds of instructions (e.g., or metaphors) or modification of the surrounding in form of restrictions or enriched environments. Thereby, a major intention of DL is to increase the possibilities of the athlete rather than constraining them. Meanwhile, evidence is provided that fluctuations that can be quantified by the amount or structure of noise can also be increased or modified by means of emotions [21] or fatigue [22,23].

Increasing the noise serves to destabilize the learning system and to launch a genuine self-organizing process. In its most extreme form, DL leads to movement variations without repetition and without correction [24]. Movement corrections in DL are avoided to enable the athlete to find their own optimal solution (which would not be the case if the athlete would be guided by information about “errors”). Moreover, external feedback can be overestimated and redundant in case of enough variation [25]. Here, it is important to differentiate augmented (or external) feedback from receiving emotional support, which could have a different influence on the training process but has not been the subject of DL research so far. The continuous confrontation with new movement challenges in DL training results in flexible and adaptable movement patterns [26,27]. According to DL theory, increased fluctuations result in better skill acquisition and better learning rates than traditional models [28,29]. Hereby, the noise should be optimized rather than maximized, which is covered by the stochastic resonance principle of the DL-theory [24,27]. Thereby, in the ideal case, the noise provided by the given exercises should be adapted to the individual and momentary noise provided by athlete. Epistemologically, the majority of studies on DL follow the strategy of conceptual replication instead of direct replication or reproduction, which logically fall too short because of the Duhem–Quine thesis, and so far provide corroboration (not verification) of the DL theory [30]. However, a differentiation of effects comparing models that were influenced and inspired by DL theory, such as the rather eclectic constraints-led approach [31] or the gradual and stochastic DL [26], are pending. Indeed, the benefits of the training programs based on DL in both technical and physical skills have been reported in team sports [32,33,34,35].

Based on the previous, including the DL approach, fluctuation in repeated sprints and sprints with COD in a training program is assumed to have the potential for eliciting physical performance improvements. Therefore, the aim of this study was to examine the effect of a four-week training intervention involving repeated differential sprint training with COD (COD) vs. without COD (NCOD) on a series of physical tests (i.e., jumping, landing, sprinting, and cutting). A better understanding of the effects of differential repeated sprint training on various aspects of physical performance may help practitioners to better schedule and design training tasks to improve these aspects. Due to the lack of comparable findings, we propose the null hypothesis, i.e., that there will be no difference in the efficacy of the repeated differential sprint training with or without COD. Furthermore, we hypothesised that repeated differential sprint training is a feasible training strategy and beneficial for physical parameters.

## 2. Materials and Methods

### 2.1. Participants

Sixteen female basketball players from the under-19 age group up to the amateur senior level volunteered in this study. All participants completed, in sum, about 270 min of basketball training (three basketball sessions/week, 90 min/session) and one to two competitive matches per week. Only participants who participated in at least 90% of the workouts were considered for data analysis, which resulted in the exclusion of seven players from post-testing analysis (NCOD, *n* = 5; COD, *n* = 4). Nine players were finally assessed. Post hoc observed power calculations (G*Power, version 3.1.9.7; University of Düsseldorf; Düsseldorf, Germany) for repeated measures ANOVA, including two groups and two measurements (α = 0.05, *d* = 0.25), revealed power (β) between 0.11 and 0.25. Written and informed consent was obtained from all participants’ parents, and player approval was obtained before the beginning of this investigation. The present study was approved by the Institutional Research Ethics Committee and conformed to the recommendations of the Declaration of Helsinki.

### 2.2. Procedures

This pilot study incorporated a parallel-groups, repeated measures design, whereby participants were randomly divided into two groups with repeated sprinting training with (COD, *n* = 8) and without COD (NCOD, *n* = 8). The training period lasted 4 weeks and was carried out within the regular training sessions. The tests were performed one and two weeks before the commencement of the training period and one week after the intervention. Physical performance tests (PPT) were performed under the same experimental conditions (training session time and indoor basketball court). A 10 min standardized warm-up was performed (5 min jogging, dynamic stretching, 10 bilateral squats, core exercises, 10 unilateral squats, and three vertical unilateral jumps) before all testing. PPTs were conducted in the following order, respecting the principles of National Strength and Conditioning Association for testing order [36]: anthropometrical measurements, jumping tests (countermovement jump (CMJ), single-leg countermovement jumps (SLCMJs)), drop jumps (DJ), single leg drop jumps (SLDJ), the 505 test, and straight sprinting tests (0–10 and 0–25 m splits time).

### 2.3. Training Program

**Figure 1:**
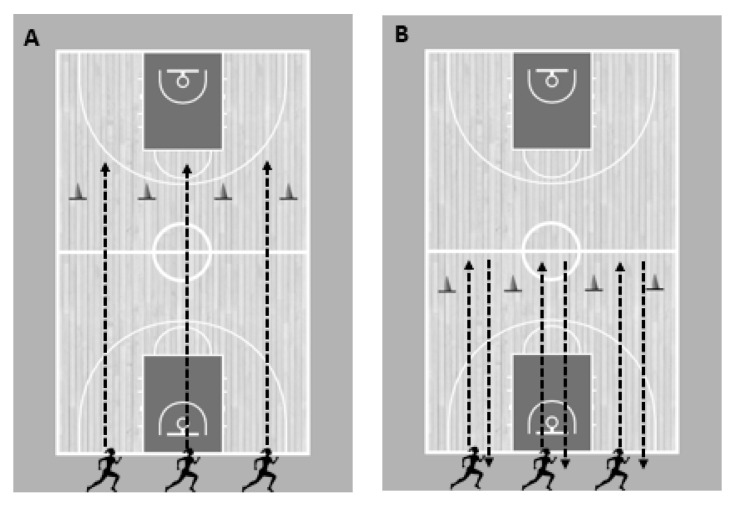
Examples of different experimental setups. (**A**) NCOD group and (**B**) COD group.

**Figure 2:**
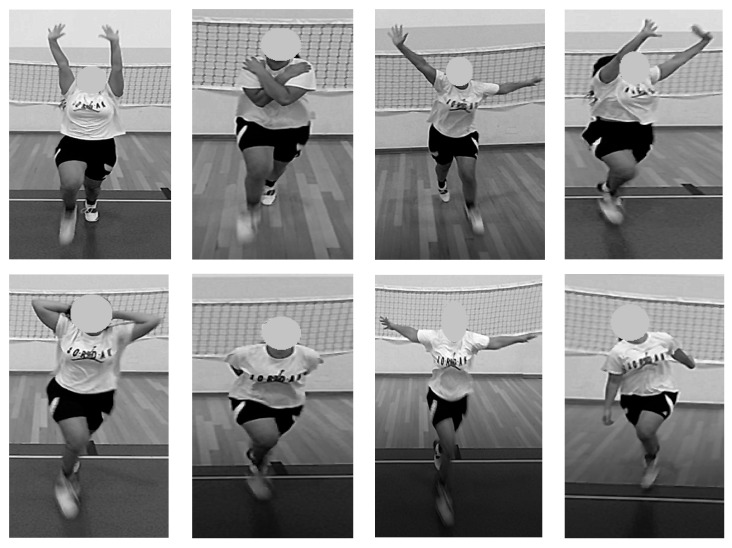
Examples of differential repeated sprint training fluctuations.

The participants included in both training groups participated in two weekly training sessions during in-court practice in a four-week period. All the intervention drills were performed at the beginning of the training session, after the warm-up period. The differential repeated sprint training was comprised by two sets of ten sprints of 20 m with 30 s of passive recovery between sprints and 3 min of passive recovery between sets. The NCOD group performed all repetitions straight, while the COD group ran to a mark situated 10 m from the starting line, performed a 180° COD using alternatively the right or left leg to push off, before returning to the starting line (total of 20 m) (Figure 1). Before each repetition, all participants were verbally instructed by the main researcher to perform a different fluctuation (Table 1; Figure 2) or a combination of fluctuations. No instructed movement fluctuation was repeated more than once in each training session. These fluctuations were selected based on previous studies involving the DL approach exercises for motor skills [28,37].

### 2.4. Measurements

Bilateral and Unilateral Countermovement Jumps (CMJ). CMJs were assessed according to the Bosco Protocol. Participants performed three successful SLCMJs with each leg in the vertical and horizontal directions. Participants began standing on one leg, then descended into a countermovement before extending the stance leg to jump as far or as high as possible in the vertical and horizontal directions. The landing was performed on both feet simultaneously. A successful trial included hands remaining on the hips throughout the movement and balance being maintained for at least 3 s after landing. If the trial was considered unsuccessful, a new trial was performed. In the horizontal direction, the participants started with the selected leg positioned just behind a starting line. The jump height was recorded using an infrared optical system (OptoJump Next—Microgate, Bolzano, Italy).

Bilateral and Unilateral Rebound Drop Jumps (DJ). Participants stood on top of a 30 cm high box with hands placed on the hips. Then, they dropped down, landed on both legs, and jumped vertically as high as possible with the shortest ground contact time possible. In unilateral rebound jumps (SLRJ), they hopped down diagonally (45° anterolateral), landed on the same leg within the infrared optical system, and then jumped vertically as high as possible with the shortest contact time possible [38]. The reactive strength index (RSI) was automatically calculated using Optojump Next software, version 1.12.1.0, through the following formula: jump height/contact time [38].

The 505 test (COD). Each participant was instructed to run to a mark situated 10 m from the starting line, perform a 180° COD using the right or left leg to push off, and return to a mark located 5 m away, covering a total of 15 m [39]. The participants were asked to pass the line indicated on the ground with their entire foot at each turn. The 505 test total time was recorded with 90 cm height photoelectric cells separated by 1.5 m (Witty, Microgate, Bolzano, Italy). Each participant performed three sprints with COD for each side with 2 min of rest between them. Players began each trial in standing positions with their feet 0.5 m behind the first timing gate. The lower limb asymmetry index (ASI) was determined by adhering to the procedures described by Bishop and colleagues [40] using the following formula: ASI = 100/Max Value (right and left)*Min Value (right and left)* − 1 + 100. The COD deficit (CODD) for the double 180° COD test for each leg was calculated via the following formula: mean double 180° COD time—mean 10 m time [39].

Speed tests. The average running speeds were evaluated by 10 m (0–10 m) and 25 m (0–25 m) split times. Running times were recorded with 90 cm high photoelectric cells separated by 1.5 m. Each participant performed three trials with 2 min of rest between each of the trials. Players began each trial in an upright standing position with their feet 0.5 m behind the first timing gate. 

### 2.5. Statistical Analyses 

Data are presented as mean ± standard deviation (SD). Reliability of test measures computed using an average measures two-way random intraclass correlation coefficient (ICC) with absolute agreement, inclusive of 90% confidence intervals, and the coefficient of variation (CV). The ICC was interpreted as poor (<0.5), moderate (0.5–0.74), good (0.75–0.9), and excellent (>0.9) [41]. Coefficient of variation values were considered acceptable if <10% [42]. A paired-samples *t*-test was used to analyse within-group changes [43]. The threshold values for Cohen’s *d* for within-group effect sizes (ES) statistics were 0–0.2 trivial, >0.2–0.6 small, >0.6–1.2 moderate, >1.2–2.0 large, and >2.0 very large [44]. A 2 × 2 repeated-measure analysis of variance (ANOVA) was performed based on the absolute values of all parameters to determine the main effects between groups (NCOD and COD group) and time (pre- and post-test) [43]. Effect size was evaluated with partial eta squared (η^2^_p_), and the threshold values were no effect (η^2^_p_ < 0.04), minimum effect (0.04 < η^2^_p_ < 0.25), moderate effect (0.25 < η^2^_p_ < 0.64), and strong effect (η^2^_p_ > 0.64) [45]. This measure has been widely cited as a measure of effect size and predominantly provided by statistical software [46]. Tukey’s post-hoc analysis was used to examine the differences between times according to THE group. All statistical analyses were performed using the SPSS software (version 24 for Windows; SPSS Inc., Chicago, IL, USA).

## 3. Results

### 3.1. Sample

The mean age of the included subjects was 19.0 years (SD: 2.4). The mean height of the subjects was 169.8 cm (SD: 5.3). The mean body mass of the subjects in this study was 62.3 kg (SD: 3.9).

### 3.2. Tests Reliability

Although the concept of systems dynamics with its essential role of fluctuations especially in phase transitions conflicts with the reliability criterium in test theory, the reliability of the chosen test diagnosis was determined for reasons of comparison and evaluation.

All ICC values ranged from moderate to excellent (ICC range = 0.54–0.93), and most (6 of the 10) of CV values were acceptable (CV range = 1.28–16.70%) (Table 2).

### 3.3. Tests Outcomes

Data from all PPTs were comparable between the two groups at baseline (all *p* > 0.05; see Table 3). 

A repeated measures ANOVA indicated a significant main effect of time on CMJ (F = 12.64; *p* ≤ 0.01; η^2^_p_ = 0.64), 0–10m (F = 13.17; *p* ≤ 0.01; η^2^_p_ = 0.65), CMJ_R_ (F = 6.43; *p* ≤ 0.05; η^2^_p_ = 0.48), and CMJ_ASI_ (F = 5.85; *p* ≤ 0.05; η^2^_p_ = 0.46). Additionally, a significant main effect of the group on SLRJ_R_ (F = 6.03; *p* ≤ 0.05; η^2^_p_ = 0.46), and CODD_R_ (F = 13.79; *p* ≤ 0.01; η^2^_p_ = 0.66) was observed. Finally, repeated measures ANOVA indicated a significant interaction effect (group x time) on CMJ_L_ (F = 12.39; *p* ≤ 0.01; η^2^_p_ = 0.64) only. The post hoc *t*-test revealed a significant difference between pre-test and post-test on CMJ_L_ for the COD training group (*p* = 0.047). 

**Figure 3:**
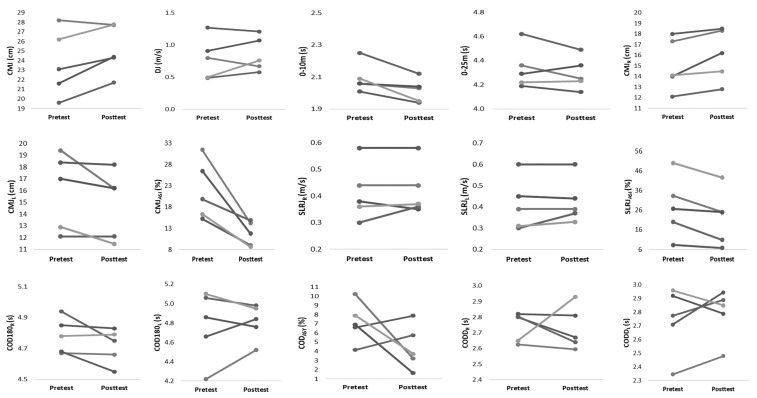
Individual changes from pretest to posttest for physical performance in NCOD group.

**Figure 4:**
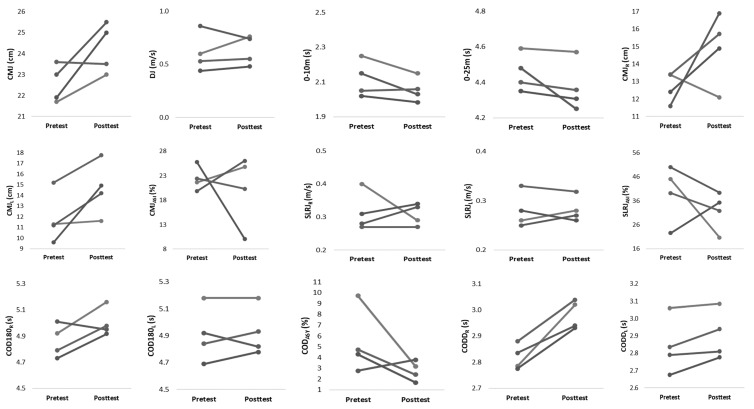
Individual changes from pretest to posttest for physical performance in COD group.

Within-group changes for both training groups are described in Table 3. The NCOD training group showed significant improvements in 0–10 m (ES = −1.25, *p* ≤ 0.05), CMJ_R_ (ES = 1.30, *p* ≤ 0.05), CMJ_ASI_ (ES = −1.86, *p* ≤ 0.05), and SLRJ_ASI_ (ES = −1.50, *p* ≤ 0.05). Figure 3 and Figure 4 display the individual changes in performance from pre- to post-test for both groups. Interestingly, the majority of athletes are responding in the same direction. Nevertheless, in both groups, single athletes can be identified who react contrary to the group trend. 

## 4. Discussion

This pilot study aimed to investigate the effects of the DL approach applied to sprint training with and without COD on physical tests in female basketball players. Methodically, in addition to the classical mean statistics, the presentation of individual results was chosen, since, on the one hand, epistemologically, from mean values only to other mean values can be concluded, but not to the individual athlete, and, on the other hand, for the effectiveness of training measures, individual reactions to interventions are increasingly of interest [47,48,49]. Considering findings and the absence of injuries and complaints, this study showed that it is feasible to implement DL principles in a repeated sprint training to improve physical performance. Moreover, the study provided indications that differential repeated sprint training has a beneficial effect on CMJ, 0–10 m, CMJ_R_, and CMJ_ASI_. Performing differential repeated sprint training without COD resulted in improved 0–10 m, CMJ_R_, CMJ_ASI_, and SLRJ_ASI_ for all group participants. Furthermore, including COD during differential repeated sprint training resulted in improved 0–25 m sprint time and CMJ_L_ for all COD group participants. 

Differential repeated sprinting training resulted in increased CMJ performance after 4 weeks. This beneficial transfer effect is similar to previous training protocols exploring DL in team sports [32,35], which suggests common underlying mechanisms explaining the improved jumping performance. Recent studies using electroencephalography to analyse brain activation patterns demonstrated neural activation after DL training in frequency bands and brain areas that are assumed to be supportive, especially for motor learning [50]. Improved motor learning and coordination (i.e., neural adaptations) dominate in the early phase of training [36]. That said, most participants may have benefited from the increased experiences of the various combinations of movement fluctuations and repeated highly intensive neuromuscular activations to improve jumping ability on the short-term scale. Specifically, increased neural drive is associated with enhanced agonist muscle recruitment, improved neuronal firing rates, and greater synchronization in the timing of neural discharge [36]. Likewise, central adaptations arising from higher neural activation can lead to increased motor unit activation, resulting in increased high-intensity muscular contraction and increased stiffness (i.e., jumping) [36]. Nevertheless, two of the participants had contrary responses (one per group) in CMJ, indicating that combining movement fluctuations and high-intensity muscular contractions places large stress either on their body or mind, inhibiting their performance. Thus, the present results need to be analysed with caution, with the need to examine the training response on an individual basis. Furthermore, practitioners should be aware that training volume, intensity, duration, or even the frequency of changes can be adjusted for negative or non-responders [51]. More studies with emphasis on individual responses are needed to better understand the effects of manipulating these variables on differential repeated sprint training [51]. 

The present findings suggest that an improvement in CMJ accompanied an increase in sprint performance. This is in accordance with the relationship between CMJ height and 0–10 m sprint time (*r* = −0.51) previously reported in female athletic populations [52]. The underlying commonality is seen in the fact that both activities take advantage of the stretch-shortening cycle (SSC), where an eccentric action (i.e., stretching) precludes a concentric action (i.e., shortening) [36]. The use of variable movements generates greater neuromuscular [53] and neurophysiological adaptations than movement repetitions [50] and increases the storage of elastic energy during the eccentric phase, leading to larger release of kinetic energy during the concentric phase. This better exploitation of the SSC may have allowed a greater training stimulus to occur over time, which, in turn, resulted in improved sprinting and jumping performance. In fact, after differential repeated sprint training, participants improved their 0–10 m sprint time. Notably, all participants of NCOD were positive responders in this test, which can be related to the specificity versus variability of the practice paradigm [54]. Possibly, the NCOD group may have benefited from better dynamic similarities between the movement patterns in differential repeated sprint training and the 0–10 m sprint test [36]. Additionally, the straight sprint and sprint with COD require specific running techniques [36], since during the COD training protocol, participants not only reduced their velocity prior to the 180° directional change at 10 m but also, due to the slowing down, caused increased eccentric contractions, while NCOD increased their velocity until 20 m.

After the differential repeated sprint training program, participants displayed higher values of CMJ_R_ compared to the pre-test values. This improvement in unilateral jumping may be indicative of increased strength of ankle- and hip-joint muscles and both static and dynamic postural balance [9]. However, to what extent these specific reactions depend on the individual preferences of the jump and play leg or laterality in general should be clarified by future research. A study analysing the effect of differential jump training on balance performance and postural control of female volleyball players during single leg stance observed a decreased sway area and anterior–posterior and mediolateral sway, indicative of improvements on the aforementioned qualities [34]. Thus, the implementation of movement variations through DL principles during training generates neuromuscular stimuli that may result in improved balance performance and ankle stabilization [55], and consequently, in improved unilateral jumping. Furthermore, previous results demonstrated impaired postural control (i.e., increased body sway) after high-intensity repeated sprints (i.e., ten 30 m sprints with two 180° COD (10 + 10 + 10 m), interspaced by 30 s of passive recovery between sprints) [56]. These findings suggest that repeated sprints result in alterations in the sensory information from the proprioceptive and exteroceptive systems [56]. Thus, continuous exposure to the combination of increased movement fluctuations accompanied by high-intensity muscular contractions can generate beneficial adaptations at the proprioceptive and exteroceptive levels that can result in positive transfer to the unilateral jumping performance in controlled settings. Notwithstanding, apart from one subject, all participants in the COD group improved CMJ_R_ and CMJ_L_ after training, including superior improvements in CMJ_L_ when compared to the NCOD group. Although the figures with individual results are no proof of individuality [48], the different effects on performance indicate that not all athletes achieved similar adaptations. 

Following the principle of stochastic resonance idea within DL-theory [28], future research should match the noise of the coach’s fluctuations to that of the athletes. Thereby, the extent to which this involves the amount or structure of noise will also need to be clarified [57]. This is particularly important in team sports since improving and strengthening all team members is crucial. The present findings suggest that the inclusion of COD during differential repeated sprint training can yet be more beneficial in unilateral jumping. Optimal movement variability during 180° COD could increase the need for lower-limb stabilisation to maintain balance, therefore requiring input from muscular involvement during triple flexion [58]. Moreover, the 180° COD involves the application of considerable braking and propulsive forces (particularly horizontal) within the final foot contact [59,60], demanding higher muscle activity of the ankle, knee, and hip-joint stabilizers. That said, improvements in unilateral jumping are expected based on the neuromuscular stimuli from simultaneously performing 180° COD with movement variation.

The overall adherence to the training programs is highly associated with the injury rate, enjoyment, motivation, and satisfaction with progress [61]. In this regard, the absence of injuries or complaints and the improvements in physical performance observed during this pilot study, in conjunction with previous adherence in experimental studies [32,35,62], bring increased expectations for a good adherence to the training program in further studies. These expectations are also sustained in brain activation patterns associated with enhanced attentional processes and improved learning processes from the use of differential learning in different motor tasks [50,63,64]. That said, continuous exposure to fluctuations can result in favorable neurophysiological adaptations, which may induce subjects to be more likely to be engaged in a training program, including differential learning approach. 

We consider this investigation a pilot study due to the small sample size arising from the dropout rate, frequently observed in youth studies [34,37]. Since we mainly rely on the original Fisher statistics, extended by the effect sizes according to Neyman–Pearson [65], there is no claim for generalization [66]. Rather, we show further supportive evidence for the effectivity of an alternative training approach that encourages self-organization within a divergent learner-oriented teaching/coaching style [67]. In agreement with the Fisher’s statistics, we conclude, based on the *p* < 0.05 results, that further research on differential training is promising. Further investigations in less-experienced, semi-professional and professional players is recommended. Furthermore, it may be worth pursuing longitudinal studies wherein the same participants are tested over months in order to fathom the complexity of the individual’s continuous changes even more [49]. The original publication on DL in 1999 was especially focused on “Individuality—a neglected parameter” to emphasize the need for alternative theoretical and practical approaches for the dominant group and average oriented streams. Longitudinal changes in each variable could help better understand the long-term training effects and further support our results. 

## 5. Conclusions

Counterintuitively, the findings of the present study displayed positive effects by adding “erroneous” fluctuation during sprint training not only in sprint but also in several jumping performances. Although it is a pilot study with no claim to generalisation, the significant changes from pre- to post-test indicate, according to Fisher’s original interpretation, that pursuing this research field is worthwhile. Whether the results due to increased noise are side or main effects demands further research. The higher variability of stimuli during the training also suggests looking for additional effects on prevention of injuries or choking in basketball of female players. This training type is recommended for further experiments in female basketball, where coaches and fitness trainers might go beyond their learned tools and switch from convergent and teacher-oriented training to more divergent thinking approaches with athlete-oriented training. Fluctuations within repeated sprint training may influence the individual’s movement patterns towards more effective and stabilized skills. From an epistemological point of view, together with all the other studies on DL, the results can be considered as a further corroboration of the DL theory. Nonetheless, much more research on the structure and amount of noise with respect to the individuality of the athletes and their momentary physiological and emotional states is necessary.

## Figures and Tables

**Table 1:** Examples of the fluctuations performed during differential repeated sprint training interventions.

#	Body Part	Fluctuations	#	Body Part	Fluctuations
1	Head	Head back	29	Trunk	Trunk rotation to the left
2		Head forward	30		Trunk rotation to the right
3		Head back and forth	31		Trunk tilted laterally to the left
4		Head rotated to the left	32		Trunk angled laterally to the right
5		Head rotated to the right	33		Torso tilted back
6		Head rotated left and right	34		Trunk tilted forward
7		Head tilted to the left	35	Hands	Hands on hip
8		Head tilted to the right	36		Right hand on hip
9	Eyes	Closed right eye	37		Left hands on hip
10		Left eye closed	38		Hands behind head
11		Blinking eyes	39		Hands on forehead
12		Look to the right	40		Right hand behind head
13		Look left	41		Left hand behind head
14	Arms	Two arms up	42		Hands behind back
15		Two arms close to the torso	43		Right hand behind back
16		Arm rotation forward	44		Left hand behind back
17		Arm rotation back	45		Clapping ahead
18		Alternate forward arm rotation	46		Clap behind back
19		Alternate backward arm rotation	47		Clap front and back
20		Arms open to the side			
21		Arms open down			
22		Crossed arms			
23		Arms stretched forward			
24		Arms stretched back			
25		Right arm up + left arm down			
26		Left arm up + right arm down			
27		Left arm up + right arm to the side			
28		Right arm up + left arm to the side			

**Table 2:** Reliability data for test variables. Data are presented as value with lower and upper confidence limits.

Test Variables	ICC (95% CL)	CV (%) (95% CL)
CMJ (cm)	0.89 (0.66; 0.97)	5.13 (2.87; 7.64)
DJ (m/s)	0.93 (0.76; 0.98)	16.70 (12.48; 20.84)
0–10 m (s)	0.81 (−0.07; 0.96)	2.63 (1.14; 4.32)
0–25 m (s)	0.84 (0.06; 0.97)	1.28 (0.46; 2.46)
CMJ_R_ (cm)	0.77 (0.19; 0.96)	10.67 (6.76; 15.07)
CMJ_L_ (cm)	0.95 (0.72; 0.99)	7.23 (4.30; 10.23)
SLRJ_R_ (m/s)	0.93 (0.63; 0.99)	14.89 (9.44; 19.37)
SLRJ_L_ (m/s)	0.95 (0.82; 0.99)	15.04 (9.43; 21.64)
COD180_R_ (s)	0.54 (−0.24; 0.89)	2.47 (1.39; 3.63)
COD180_L_ (s)	0.86 (−0.12; 0.97)	2.71 (1.35; 4.18)

Abbreviations: ICC = intraclass correlation coefficient; CV = coefficient of variation; CL = confidence limits; CMJ = countermovement jump height; DJ = drop rebound jump; 0–10 m = 0–10 m sprint time; 0–25 m = 0–25 m sprint time; SLRJ = diagonal single leg rebound jump; COD180 = change of direction test; R = right; L = left.

**Table 3:** Inferences of the training programs intervention on player’s performance measures.

Variables		Pretest, Mean ± SD	Postest, Mean ± SD	*p*	Within-Group Effect Size	Between-Groups Pretest Differences (*p*)	Between-Group Effect Size
CMJ (cm)	NCOD	23.74 ± 3.47	25.17 ± 2.58	0.061	1.16	0.530	0.44
	COD	22.55 ± 0.90	24.53 ± 1.19	0.095	1.21		
DJ (m/s)	NCOD	0.79 ± 0.32	0.86 ± 0.27	0.418	0.40	0.340	0.67
	COD	0.61 ± 0.18	0.63 ± 0.14	0.686	0.22		
0–10 m (s)	NCOD	2.09 ± 0.09	2.02 ± 0.07	0.035 *	−1.25	0.730	−0.24
	COD	2.12 ± 0.10	2.06 ± 0.07	0.132	−1.03		
0–25 m (s)	NCOD	4.34 ± 0.17	4.29 ± 0.13	0.322	−0.61	0.266	−0.81
	COD	4.46 ± 0.10	4.37 ± 0.14	0.183	−0.86		
CMJ_R_ (cm)	NCOD	15.10 ± 2.47	16.05 ± 2.45	0.044 *	1.30	0.110	1.22
	COD	12.70 ± 0.87	14.90 ± 2.04	0.202	0.82		
CMJ_L_ (cm)	NCOD	15.96 ± 3.28	14.84 ± 2.91	0.122	−0.87	0.074	1.41
	COD	11.83 ± 2.38	14.61 ± 2.53	0.073	1.36		
CMJ_ASI_ (%)	NCOD	21.86 ± 6.93	11.68 ± 2.87	0.014 *	−1.86	0.881	−0.10
	COD	22.43 ± 2.50	20.30 ± 7.25	0.691	0.22		
SLRJ_R_ (m/s)	NCOD	0.43 ± 0.12	0.42 ± 0.10	0.672	−0.20	0.123	1.18
	COD	0.32 ± 0.06	0.31 ± 0.03	0.847	0.11		
SLRJ_L_ (m/s)	NCOD	0.42 ± 0.12	0.43 ± 0.10	0.859	0.09	0.063	1.48
	COD	0.32 ± 0.06	0.31 ± 0.03	0.073	−0.10		
SLRJ_ASI_ (%)	NCOD	27.67 ± 15.53	22.01 ± 14.10	0.028 *	−1.50	0.262	−0.82
	COD	39.19 ± 11.92	31.80 ± 8.03	0.405	−0.48		
COD180_R_ (s)	NCOD	4.78 ± 0.11	4.76 ± 0.17	0.703	−0.18	0.361	−1.00
	COD	4.86 ± 0.13	5.00 ± 0.11	0.131	1.03		
COD180_L_ (s)	NCOD	4.78 ± 0.36	4.80 ± 0.18	0.824	0.10	0.550	−0.42
	COD	4.91 ± 0.21	4.93 ± 0.18	0.690	0.22		
COD_ASY_ (%)	NCOD	7.16 ± 2.19	4.44 ± 2.43	0.196	−0.69	0.336	0.69
	COD	5.38 ± 3.02	2.77 ± 0.92	0.190	−0.85		
CODD_R_ (s)	NCOD	2.74 ± 0.09	2.73 ± 0.14	0.895	−0.06	0.176	−1.01
	COD	2.82 ± 0.05	2.98 ± 0.06	0.009 **	3.06		
CODD_L_ (s)	NCOD	2.74 ± 0.24	2.79 ± 0.18	0.533	0.30	0.515	−0.46
	COD	2.84 ± 0.16	2.90 ± 0.14	0.074	1.35		

Abbreviations: CMJ = countermovement jump height; DJ = drop rebound jump; 0–10 m = 0–10 m sprint time; 0–25 m = 0–25 m sprint time; SLRJ = diagonal single leg rebound jump; COD180 = change of direction test; CODD = COD deficit; R = right; L = left; ASI = bilateral asymmetry; * significant differences at *p* < 0.05; ** significant differences at *p* < 0.01.

## Data Availability

The data that support the findings of this study are available from the corresponding author, J.A., upon reasonable request.
